# Water Access, Sanitation, and Hygiene Conditions and Health Outcomes among Two Settlement Types in Rural Far North Cameroon

**DOI:** 10.3390/ijerph14040441

**Published:** 2017-04-20

**Authors:** Tyler J. Gorham, Joshua Yoo, Rebecca Garabed, Arabi Mouhaman, Jiyoung Lee

**Affiliations:** 1 Division of Environmental Health Sciences, College of Public Health, The Ohio State University, Columbus, OH 43210, USA; gorham.20@osu.edu; 2 Department of Veterinary Preventive Medicine, The Ohio State University, Columbus, OH 43210, USA; yoo.205@osu.edu (J.Y.); garabed.1@osu.edu (R.G.); 3 Department of Environmental Sciences, The Higher Institute of the Sahel, University of Maroua, Maroua BP 46, Far North Region, Cameroon; mouharabi@yahoo.fr; 4 Department of Food Science and Technology, The Ohio State University, Columbus, OH 43210, USA

**Keywords:** microbial source tracking, antibiotic resistance, enteric pathogens, diarrhea disease, health behavior

## Abstract

The Far North region in Cameroon has been more heavily impacted by cholera than any other region over the past decade, but very little has been done to study the drivers of waterborne diseases in the region. We investigated the relationship between water, sanitation, and hygiene (WASH) parameters, microbial and antibiotic resistance (AR) contamination levels in drinking water, and health outcomes using health survey and molecular analysis during June and July of 2014 in two settlement types (agro-pastoralist villages and transhumant pastoralist camps). Quantitative polymerase chain reaction was used to determine fecal contamination sources, enteric pathogens, and antibiotic resistance genes. Ruminant-associated fecal contamination was widespread in both settlement types (81.2%), with human-associated contamination detected in 21.7% of the samples. *Salmonella* spp. (59.4%) and Shiga toxin-producing *E. coli* (*stx*1 44.9% and *stx*2 31.9%) were detected across all samples. Tetracycline resistance was found only in village samples. A significant difference in diarrheal incidence within the past 28 days among young children was found between camps (31.3%) and villages (0.0%). Our findings suggest that water contamination may play an important role in contributing to gastrointestinal illness, supporting the need for future research and public health intervention to reduce gastrointestinal illness in the area.

## 1. Introduction

Diarrheal illness was the underlying cause of roughly 1.5 million deaths globally in 2012 [1]. In low and middle income nations, inadequate access to safe water, poor hygiene, and unimproved sanitation conditions (also known as the WASH paradigm) are responsible for 502,000 deaths per year: 58% of all diarrheal disease-related deaths in these countries [1]. Much of this burden is the result of diarrheal illness, as poor WASH conditions contribute to the spread of gastrointestinal pathogens via mainly the fecal-oral route [2]. Concerning microbial water quality, the largest contributor to illness is fecal contamination of drinking water and its sources (thereby adding enteric pathogens). Breaking this transmission cycle requires improvements in hygiene (such as handwashing behaviors), sanitation facilities, and/or improved drinking water sources and treatment.

Fortunately, high-quality WASH interventions have proven to be effective in combatting diarrheal illness. A recent meta-analysis found that hygiene-based interventions, such as hygiene education campaigns and handwashing promotion, may reduce the risk of diarrheal illness by 45%, with a pooled estimated relative risk of 0.55 (95% CI 0.40–0.75) [3]. However, this protective effect may be reduced in settings with limited access to water [4]. Further, household, point-of-use water treatment (boiling, chlorination, etc.) in rural areas may reduce diarrheal illness by 39% [3]. Improved drinking water supplies (such as public taps, boreholes, protected springs, or water piped directly to homes) may help reduce the transmission of enteric pathogens through drinking water treatment or source water protection, but randomized trials studying the effectiveness of water supply interventions are limited [3,4]. Unfortunately, of the 663 million people still lacking access to an improved source of drinking water, nearly half live in sub-Saharan Africa [5].

Over the duration of the Millennium Development Goals (MDGs) (1990–2015), access to improved water sources in rural Cameroon improved from just 34% coverage to 53% [6]. Access to improved or shared sanitation sources increased by just 3% from 1990 to 2015 in urban Cameroon (from 82% to 85%) and did not improve at all in rural areas of the country, remaining at an estimated 27% throughout the MDG timeframe [6]. One improvement made in rural areas is the decrease in open defecation from 17% to 12% over the 25-year period [6]. The combination of low access to safe water and poor sanitation conditions is made evident by Cameroon’s recent history of cholera outbreaks and the burden of disease attributed to diarrheal illness in the country, especially in the Far North region [7,8].

Despite Cameroon’s greatest cholera outbreaks often occurring in the Far North region [7], the majority of water quality studies conducted in Cameroon have taken place outside of this region, near Cameroon’s two largest cities, Yaoundé and Douala [9,10,11,12,13,14]. This disproportionate impact of cholera on the Sahel region is not specific to Cameroon, as Chad’s Guera region reported 1754 cases of cholera during the outbreak in 2011 [15]. Closer to the Far North, Djaouda et al., measured high and consistent levels of fecal contamination in wells used as a source for drinking water in the North region of Cameroon [16,17]. Working in the Logone Valley, at the border of Chad and Cameroon, Sorlini et al., studied drinking water sources in the Far North and noted that current World Health Organization (WHO)/United Nations International Children’s Emergency Fund (UNICEF) Joint Monitoring Program for Water Supply and Sanitation (JMP) definitions of “improved sources” may not actually be protective of human health [18,19]. Recently, Healy Profitós et al. investigated water quality in Maroua, the capital city of the Far North, offering insight on water quality degradation across informal distribution systems and health outcomes in an urban setting [20], as well as a recent microbial source tracking and pathogen detection investigation [21]. A significant gap remains in the literature of studies simultaneously studying WASH conditions and health outcomes in rural Far North, Cameroon, particularly with respect to nomadic populations.

The current study seeks to first survey the status and relationship between factors of WASH and diarrheal illness among agro-pastoralists living in established villages and transhumant pastoralists living in nomadic camps in rural Far North, Cameroon. The results of this study not only serve as a baseline understanding of these issues in the rural Far North, but also provide insight into the differences between these two rural study populations and those living in the urban center of Maroua. The outcomes of this study can provide a better understanding for future public health initiatives directed at improving WASH conditions in Far North, Cameroon, such as increased access to improved drinking water sources and water handling practices for transhumant pastoralists.

## 2. Materials and Methods

### 2.1. Study Site

**Figure 1:**
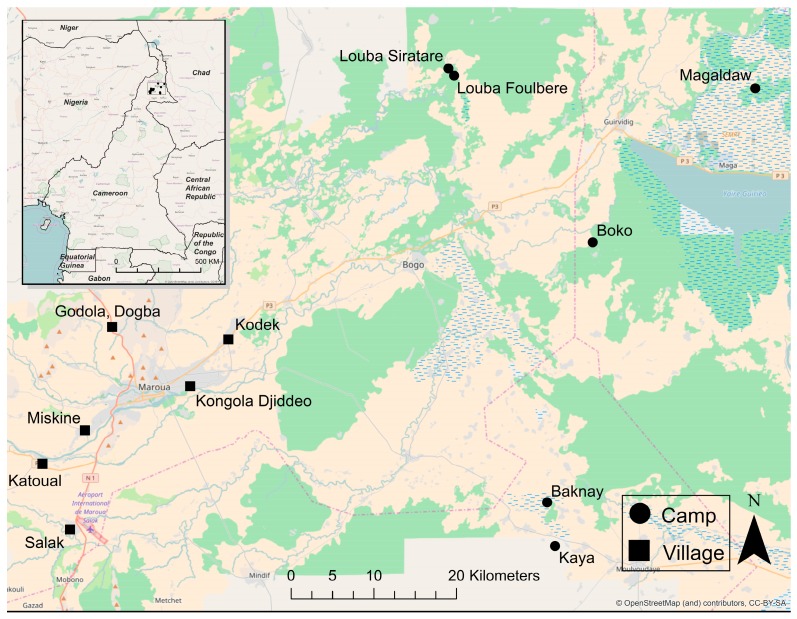
Locations of health survey administration and water sampling, over June and July 2014. Six transhumant pastoralist camps and six sedentary villages within a triangle formed by the cities of Maroua, Yagoua, and Kaele were arbitrarily selected for sampling.

The study was conducted in the Far North of Cameroon, where six transhumant pastoral camps (Boko, Baknay, Kaya, Magaldaw, Louba Foulbere, Louba Siratare) and six permanent agro-pastoralist villages (Meskine, Salak, Katoual, Godala, Kodek, Kongola) are within a triangle formed by the cities of Maroua, Yagoua, and Kaele (Figure 1). The sites were arbitrarily selected. The climate is Soudan Sahelian type and the vegetation is characterized by savanna shrubs and graminacea. A climate gradient is observed in the region between the dryer areas in the North and those in which a more humid Sudanese climate prevails towards the south. Soils are predominantly made of vertisols, alluvial and hydromophic soils in the flood plain, whereas washed tropical soils, ferriginous soils, fersialitic and lithosoils are prevalent in the dry part. Most water used for drinking and domestic activities comes from wells or community boreholes and is mainly stored in canaries (wide-mouth clay jars) or jerry cans [20]. Water sampling and survey implementation were conducted between June and July 2014, during the end of the rainy season. The areas are flat with a dry plain to the west and a floodplain to the east.

Two agricultural systems are practiced among the study sites: agro-pastoralism and transhumant pastoralism. Residents of established villages practice agro-pastoralism, relying primarily upon crop farming, including cotton, millet, peanuts, and vegetables. In this area, it is also common for families to have at least one cow, though livestock production is not a primary source of income. In nomadic camps, individuals practice transhumant pastoralism, where livestock rearing is the only source of income and herders travel with their cattle to allow for open grazing. Generally, these pastoral communities are seasonal migrants, traveling from the Kaele area in the south in the rainy season to the Logone Floodplain in the north of the region during the dry season as the flood water recedes.

### 2.2. Water Sampling

Samples of household water and the reported drinking water source (when available; N = 70) were collected from survey participants into sterile, 800 mL Whirl-Pak bags (Nasco, Fort Atkinson, WI, USA). Among villages, 15 samples were collected from drinking water sources and 29 samples were collected from homes, while 6 water samples were collected from nomadic camp drinking water sources and 20 were collected from camp homes (Figure 1). Household water—used for drinking, cooking, and washing—was collected directly from the household’s storage container(s). In the case of a central household water storage vessel (such as a canari), the samples were collected using the same method that the residents use to serve water, such as collecting water from the canari using a shared cup or ladle. Source water was collected per the study participants’ main water source, which ranged from surface water in many camps to drilled boreholes in villages. After collection, between 40 and 500 mL of water sampled was concentrated via membrane filtration onto 0.45 µm Millipore membranes (IsoporeTM Membrane Filters, Millipore, Tullagreen, Cork, Ireland) using a manual syringe. The range in the volume of water concentrated was due to membranes clogging before 500 mL could be passed through for highly turbid samples. The total amount filtered was recorded. After concentration, sample storage and transport were conducted as reported by Healy Profitós et al. [20].

### 2.3. Molecular Analysis

The filtered membranes were kept frozen and then transported on ice to the lab at The Ohio State University (Columbus, OH, USA). To begin DNA extraction, each membrane was transferred into a sterile 2 mL microcentrifuge tube containing 1.4 mL of ASL buffer (provided in the QIAmp DNA Stool Mini Kit; QIAGEN, Valencia, CA, USA), as well as sterile glass beads 0.1 mm and 0.5 mm in diameter. Bead-beating was then performed for 3 min at 2100 oscillations per minute in a Mini-Beadbeater-96 (Biospec Products, Bartlesville, OK, USA). After bead-beating, the supernatant was transferred to sterile 2 mL microcentrifuge tubes and DNA was extracted using a QIAamp DNA Stool Mini Kit, following the manufacturer’s instructions (QIAGEN, Valencia, CA, USA).

The presence of quantitative PCR (qPCR) inhibitors in water samples was investigated using Sketa22 qPCR assay [22]. None of the extracted DNA samples demonstrated the presence of PCR inhibitors. In order to investigate sources of water contamination, microbial source tracking was conducted by targeting two human-specific genetic markers and one specific to ruminant fecal contamination. Human markers included: HF183, a 16S rRNA gene of human-specific *Bacteroides* spp. [23,24] and *gyr*B, a target gene of gyrase subunit B from human-specific *Bacteroides fragilis* [25,26,27]. Rum2Bac, a *Baceroidales* 16S rRNA gene, was chosen for its ruminant gut microbiome specificity [28].

Quantitative PCR was also used to investigate the presence and concentrations of pathogens both in household water and drinking water source. Virulence factor genes for Shiga toxin types 1 and 2 (*stx*1 and *stx*2, respectively), from Shiga toxin-producing *Escherichia coli* (STEC) were tested [29]. A second group of pathogens also associated with fecal contamination of water, *Salmonella* spp., were investigated using a 16S rRNA gene target [30]. Lastly, as a preliminary investigation of antimicrobial resistance gene presence, resistance to tetracycline was tested by targeting *tet*Q [31]. Resistance to tetracycline was chosen among the wide variety of antibiotics based on informal observation of local practices during sampling in our previous study of water quality in the region [20]. All qPCR analyses were conducted using a Bio-Rad CFX96 Touch thermocycler (Bio-Rad Laboratories, Inc., Hercules, CA, USA).

### 2.4. Health Survey

For each settlement selected (with the exception of a single camp, Lauba Siratare), in-home health surveys were administered, as conducted by Healy Profitós et al. [20]. Necessary research permissions were obtained through The Ohio State University’s Institutional Review Board (IRB 2010B0004) and Dr. Arabi Mouhaman’s status as faculty at the University of Maroua (Law No. 005 of 16 April 2001; Decree No. 93/035 of 19 January 1993 on the special status of the higher education personnel). Twenty-five village household surveys (including 159 individuals) and 14 camp household surveys (including 86 individuals) were collected during June and July of 2014. Topics covered in the survey included demographics, household-level hygiene behaviors (e.g., handwashing practices), access to improved sanitation, water source and accessibility, and recent health status, including diarrheal incidence. Household water source, water storage vessel type, hygiene behavior, and access to sanitation facilities were surveyed at the household level, but responses were assigned to individual respondents, based on head of household response. This allowed for comparison to national and global rates by accounting for the percentage of the study population, rather than percentage of households. Consent and privacy considerations were handled as previously reported [20].

### 2.5. Statistical Analysis

Exploratory data analysis and statistical analyses were conducted using RStudio Version 0.98.1103 (RStudio, Boston, MA, USA; R version 3.2.4, R Core Team, Vienna, Austria); tables and descriptive pie charts were generated in Excel 2016 (Microsoft, Redmond, WA, USA). The R package ggplot2 was used to create bar plots [32]. Before analysis, gene copy results from qPCR were log-transformed to allow for a more normal distribution of gene copy concentrations and values detected-but-not-quantifiable (DNQ) were set to one-half the limit of quantification [21]. Further, three of the samples needed to be eliminated from the dataset due to uncertainties in recorded filtered volumes or status as Home or Source water samples, resulting in 67 water samples with qPCR results available for analysis. A Shapiro-Wilk test determined the molecular data to be non-normal, with the exception of *tet*Q. Because of the non-normal nature of the molecular data, comparisons between gene copy numbers among sample types were conducted using the Wilcoxon Rank Sum test. Chi-square tests, risk ratios, Fisher’s exact test, and the Cochran-Mantel-Haenszel adjusted odds ratios were conducted by comparing survey results among different settlement types.

## 3. Results

### 3.1. Population Demographics

Demographic data from the surveys are presented in Table 1. Though not statistically significant, camp populations were slightly younger, with 20% of their population being under 5 years old (compared to just 9% in villages). The average household size among villages and camps were 6.36 and 6.14, respectively. It is important to note that not all questions were answered by all respondents, as some respondents preferred to not answer certain questions, so summary statistics may not reflect all 245 surveys collected.

### 3.2. Microbial Water Quality and Pathogen Analysis

**Figure 2:**
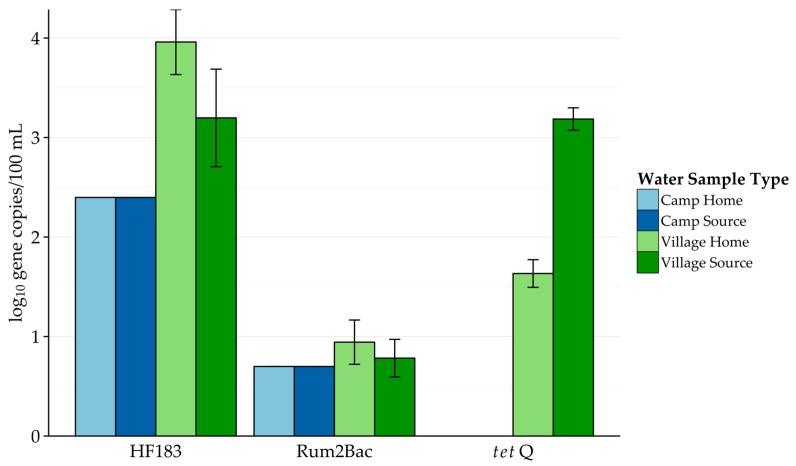
Concentration of microbial source tracking (MST) and antibiotic resistance (AR) genes by water sample type. Among the samples detected, HF183 was present at much higher concentrations than Rum2Bac. *tet*Q was only detected in village samples. Error bars represent the standard error of the mean.

**Figure 3:**
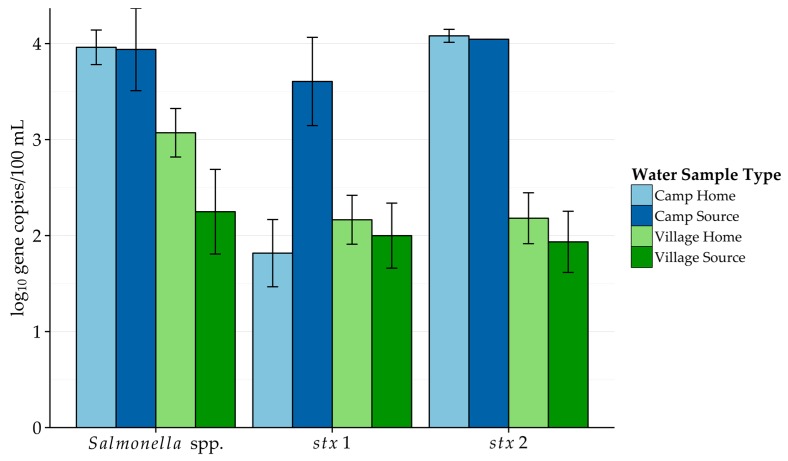
Concentrations of pathogens detected by quantitative PCR (qPCR) in different water sample types (camp vs. village and water at home vs. water from the source water). The drinking water from the source at camps showed the highest concentrations of pathogens. Among villages, water from homes had slightly higher contamination levels, whereas source water contamination levels were higher in camps. Error bars represent the standard error of the mean.

Results of the molecular analysis are presented in Table 2, Figure 2 and Figure 3. Overall, Rum2Bac (ruminant specific) was consistently detected at low levels. While the HF183 (human specific) was detected in each of the sample types, *gyr*B was not detected anywhere. When detected, HF183 was present at higher concentrations than Rum2Bac, but was detected at a much lower frequency. Among the pathogens, *Salmonella* spp. was detected more frequently than either STEC marker, across all sample types, and at higher concentrations in both village sample types. Finally, *tet*Q was only detected in water collected from villages.

Drinking water sources among camps showed the highest frequency of detection for both human and ruminant fecal markers. Further, ruminant fecal contamination was frequently detected across sample types. Village water samples were the only ones to contain tetracycline-resistance genes.

### 3.3. Water Sources, Storage and Disinfection

**Figure 4:**
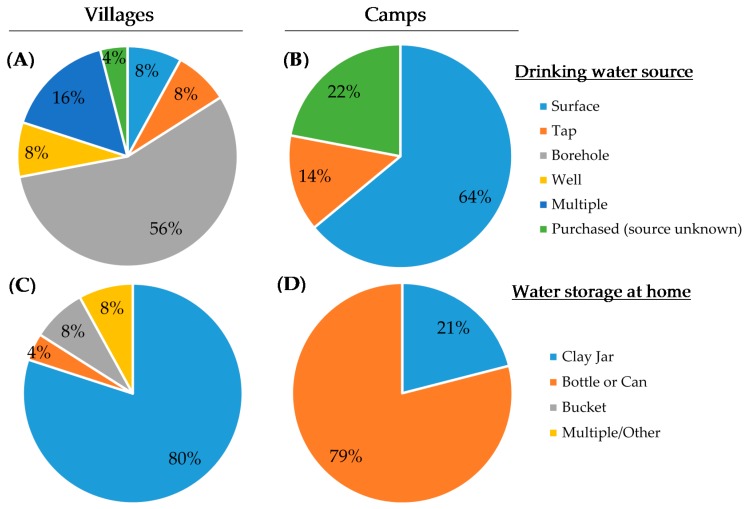
Water storage methods and reported primary sources of drinking water. Village residents reported high borehole reliance (**A**), while those in transhumant pastoralist camps relied primarily on surface water (**B**). For water storage, most village residents use clay jars (**C**) while nomadic camp residents rely heavily on bottles and jerry cans (**D**).

Among camps, 64% of individuals reported relying on surface water for their primary household water source (Figure 4B). Fourteen percent of camp households surveyed were served by tap water, but the status of these taps as boreholes or improved water sources is unknown. Further, 22% of individuals in camps purchase water and the original source of this water is also unknown. A wider variety of water sources were reported among those living in established villages (Figure 4A), categorized during analysis as the following: surface water sources, taps, boreholes, wells, multiple sources, and again purchased sources. Among villages, boreholes, taps, and wells composed 72% of participants’ water sources. Differences in water storage containers were also seen between camps and villages, with 79% of individuals relying on bottles or cans for water storage in nomadic camps and 80% of individuals using clay jars in villages (Figure 4C,D). Regarding disinfection of water with chlorine (as bleach), 17% of village households report always treating water with chlorine, with 54% reporting occasional bleach use. These rates were lower in camps, where only 7% of households reported always using bleach and an additional 43% report sometimes using bleach to treat water before consumption.

### 3.4. Sanitation and Hygiene

Differences in open defecation and hygiene practices were also seen between camps and villages at the household and individual levels. While only 2.6% of individuals practice open defecation in villages, roughly 19% do so in camps (Table S1). Outdoor latrine use rates were similar between villages and camps, at 79% and 81%, respectively. As would be expected given the portable nature of nomadic camps, no indoor sanitation facilities were reported in this population, while 18% of individuals in villages reported primary use of indoor sanitation facilities. Lastly, while trends in reported handwashing rates seemed to be apparent, significant differences were not found between villages and camps (Table S2).

### 3.5. Health Surveys

Three major gastrointestinal illnesses surveyed (within the past 28 days) were diarrhea or loose stools, diarrhea or loose stools with blood, and stomach cramps (Table 3). No significant differences (α > 0.05) were seen at the population level in these rates between camps and villages (Table S3). When comparing diarrheal rates by age group, a significant difference was found between camps and villages among children under 5 years of age (Table 3). Diarrheal incidence within the past 28 days was 0% for children aged 0–4 among villages, but was 31.3% among children in camps.

## 4. Discussion

### 4.1. Rural WASH Conditions

The results of the drinking water source highlight the importance of studying the status of local WASH conditions. Stark differences were seen regarding reliance on surface water between villages and nomadic camps. Comparing the WHO/UNICEF Joint Monitoring Program’s [6] estimate of 16% of rural Cameroonians relying on surface water to 64% (camps) and 8% (villages) from this study, one can see the importance of understanding the resources of local communities. Being nomadic, transhumant pastoralists must rely on more easily accessible water sources for themselves and their cattle, which is likely the reason for such a high reliance on surface waters. Water storage trends also reflect this difference in lifestyle, as the jerry cans and bottles used by homes in nomadic camps would be much more portable than the clay jars used by most village homes.

Regarding sanitation and hygiene, general trends suggest worse conditions in camps than in villages. A relatively high open defecation rate in camps (19%) suggests that a difference in access to proper sanitation may exist. The open defecation rate of 4% in villages is much lower than the WHO/UNICEF Joint Monitoring Program’s estimate of 12% of the rural Cameroon population [33]. Though the differences were not statistically significant, it appears from our study that reported handwashing is more common in villages than camps, especially handwashing with soap (Table 2).

A few major trends can be seen in the molecular analysis of the region’s water quality. First, fecal contamination of water sources by ruminants was very persistent, with detection rates ranging from 65% to 100%. Interestingly, despite higher open defecation rates, human fecal contamination levels appeared to be only slightly higher in camps than in village source water, and were lower among household water samples. The presence of *tet*Q in villages alone may suggest differences in antibiotic use (for humans and/or animals) in the two settings.

STEC are common zoonotic pathogens associated with fecal contamination from animals (especially cattle) and humans; Shiga toxins are capable of causing gastrointestinal illnesses ranging from diarrhea to the potentially fatal hemolytic uremic syndrome in humans [34]. Accordingly, Shiga-toxin producing genes *stx*1 and *stx*2 were studied to assess the potential presence of Shiga-toxin producers. *Salmonella* were also investigated due to the importance of Typhoid fever in the region and the recent identification of this genus from a wide variety of water sources in the Logone Valley, which includes Far North, Cameroon [19]. Thus, *Salmonella* spp. was tested as a screening tool for potential presence of *Salmonella*-related pathogens. No clear pattern was seen in pathogen detection rates when comparing villages to camps. However, when considering the contamination level among those samples that were positive, camp water sources had higher *stx*2 and *Salmonella* spp. gene copy numbers. These results may be taken together to say that while source and household water in camps was not more frequently contaminated by *Salmonella* spp. and Shiga toxin-producing *E. coli*, these water samples had higher contamination levels of these pathogens than similar village water samples.

Considering the impact of WASH characteristics on gastrointestinal illness, one would expect the impact of higher contamination levels in water consumed by individuals in nomadic camps to only be worsened by the lower reported handwashing rates. This effect was not shown at the population level, as diarrheal rates were not significantly different between populations in camps and villages. However, a significant difference in diarrheal incidence was seen among children under 5 years of age when comparing camps to villages (31.3%, 0%; *p*-value < 0.05). This is an important finding when considering the impact that diarrheal illness has on the global under 5 mortality rate. Children’s immune systems have not yet adapted to the pathogens encountered in day-to-day life. Because of this, this difference in diarrheal rates may act as an indicator of differences in WASH conditions, whereas the stronger immune systems of adults who have adapted to their individual settings may dilute this effect. Oloruntoba, et al. found improper water handling and insufficient caretaker handwashing to be significant contributors to diarrheal risk among children under five in Ibadan, Nigeria. These may present important intervention points in the reduction of the under-5 diarrheal rate in rural Far North, Cameroon, but additional studies on handwashing behaviors are needed [35]. Additionally, we measured higher gene concentrations for all three pathogen markers studied in camps as opposed to villages (though not statistically significant), which may also contribute to this difference.

Worth noting, other factors may be driving this difference in diarrheal rates, such as access to medical care to prevent reoccurring illnesses and differences in direct transmission in different households. Additionally, children in nomadic villages are likely in closer proximity to cattle and, therefore, they might have more chances to get exposed to cattle waste when they play outside. This direct contact with fecal pathogens may also contribute to differences in the under-5 diarrheal rates observed. Though not statistically significant, village residents appeared to visit hospitals more frequently, with 14% of respondents having visited a hospital within the past 30 days, compared to just 6% of camp residents. Further, a significant difference was not found between the proportions of respondents having been under the care of a physician between camps (7%) and villages (10%).

### 4.2. Comparison with Urban Water Quality in Far North, Cameroon

This study serves as a follow-up to previous work conducted by Healy Profitós et al. [20,21] who investigated water quality along informal water distribution systems in urban Maroua, Cameroon. Across both rural camps and rural villages, evidence of human fecal contamination suggests rural areas are subject to greater human impacts on microbial water quality, when compared to the urban area of Maroua. While only 7% of urban water samples collected showed HF183 detection [20], the same marker was detected in 20%–33% of rural samples in this study (Table 2). Comparing the detection rates of *tet*Q, rural villages had the highest *tet*Q detection rate among all sample types (47%), urban source water samples were second highest (28%), and similar rates were found among urban homes (13%) and rural village homes (14%). Note that no tetracycline resistance genes were found in the rural camp water samples.

Concerning non-bloody diarrheal rates, the rate among children was lower in rural villages than in Maroua among children under five years old (0% and 23.7%, respectively) [20]. However, rural camps reported the highest diarrheal rates in this age group, at 31.3%. Diarrheal rates were higher for children 5 years and older in Maroua, as compared to rural camps and villages (11.9%, 0%, and 1.7%, respectively) [20]. Among adults, non-bloody diarrheal rates were similar across all study areas.

### 4.3. Study Limitations and Future Work

Study limitations primarily pertain to the scale of this study. Surveys and water samples were collected as a small portion of a larger study in Cameroon, so resources were limited. This factor surfaced regarding the surveys, as respondent numbers were not equal between camps and villages, and in the collection of water samples, where small sample numbers likely contributed to insignificant findings, though trends were observed. Questionnaire response rates were strong, but item non-response on the surveys may present a bias that we were unable to account for. Further, behavior and health outcome measurements in this study relied on self-reported health history and hygiene behavior survey questions. These self-reported data are subject to both recall bias and potential misreporting, where negative perceptions of illness or improper hygiene behaviors may influence survey participants’ responses.

Another limitation of this study pertains to the number of microbial source tracking markers and antibiotic resistance genes investigated. To sufficiently investigate the sources of fecal contamination, further analysis of human- and ruminant-specific markers would be helpful, as geographic difference in gut microbiomes may exist. Thus, an investigation of the best host-specific fecal markers should be conducted, but this analysis was beyond the scope of our study. As mentioned, tetracycline resistance genes were investigated as a snapshot of antibiotic resistance, but tetracycline is not the only antibiotic used in this region for humans or animals. A known limitation of using molecular techniques to study pathogens is that these techniques test for the presence of genes rather than viable organisms; thus, the presence/levels of detected target pathogens should not be directly interpreted as a known risk for pathogen infection.

It is important to note that the villages under study, while rural, are near the provincial capital of Maroua. Given this, our study may underestimate the need for improved WASH conditions in rural villages of Cameroon, as areas just outside of cities are likely to have more access to healthcare services and WASH essentials, such as chlorine and soap.

## 5. Conclusions

Fecal contamination from ruminants and humans was common throughout the study area. This was reflected in the detection of *Salmonella* spp. and STEC by qPCR across both source water and drinking water sample types. Open defecation is likely a contributing factor to the fecal contamination of water sources. While hygiene practices were not significantly different between camps and villages, diarrheal disease rates among children under five were found to be higher in nomadic camps. The culmination of these findings sheds light on the great amount of progress needed toward clean water access and improved sanitation and hygiene conditions in rural Far North, Cameroon.

## Figures and Tables

**Table 1:** Population Demographics by Community Type.

	Village	Camp
	Count (%) *	Count (%) *
**Age Group**		
Under 5	15 (9)	16 (20)
5–17	60 (38)	28 (34)
18–60	72 (46)	35 (43)
60+	11 (7)	3 (4)
**Sex**		
Male	73 (46)	42 (50)
Female	86 (54)	42 (50)

* Age and sex data not collected for every survey respondent; totals do not sum to total survey number (*n* = 245). The two community types show very similar demographics. Camps had a slightly younger population compared to villages.

**Table 2:** Target gene detection frequency by water sample source.

Water Type	HF183	Rum2Bac	*tet*Q	*stx*1	*stx*2	*Salmonella* spp.
**Village Source**	20.0%	93.3%	46.7%	46.7%	46.7%	60.0%
**Village Home**	21.4%	82.1%	14.3%	50.0%	35.7%	53.6%
**Camp Source**	33.3%	100.0%	0.0%	50.0%	33.3%	33.3%
**Camp Home**	20.0%	65.0%	0.0%	35.0%	15.0%	65.0%

**Table 3:** Incidence of reported gastrointestinal health conditions in past 28 days by age group. There was an apparent difference in diarrheal rates (bloody and non-bloody) between camps and villages among adults aged 60 years and older, but this is based on only three individuals in camps.

Health Condition	% Village (*n*)	% Camp (*n*)	Fisher’s Exact Test *p*-Value
**Non-Bloody Diarrhea**			
Under 5	0 (15)	31.3 (16)	0.04 *
5–17	1.7 (60)	0 (27)	1.00
18–60	4.2 (72)	8.6 (35)	0.39
60+	9.1 (11)	0 (3)	1.00
**Bloody diarrhea**			
Under 5	0 (15)	12.5 (16)	0.48
5–17	1.7 (59)	0 (27)	1.00
18–60	0 (72)	2.3 (35)	0.33
60+	9.1 (11)	0 (3)	1.00
**Stomach cramps**			
Under 5	6.7 (15)	12.5 (16)	1.00
5–17	13.3 (60)	7.1 (28)	0.49
18–60	18.3 (71)	8.6 (35)	0.25
60+	20.0 (10)	0 (3)	1.00

* *p*-value < 0.05.

## Supplementary Materials

The following are available online at www.mdpi.com/1660-4601/14/4/441/s1, Table S1: Open defecation rate and hygiene facility availability in the study sites, Table S2: Household-level Handwashing Rates reported during the study period, Table S3: Incidence of reported gastrointestinal illnesses in the past 28 days.
