# Supplementary material for: Water Access, Sanitation, and Hygiene Conditions and Health Outcomes among Two Settlement Types in Rural Far North Cameroon
Source: Int J Environ Res Public Health. 2017 Apr 20;14(4):441. doi: 10.3390/ijerph14040441 (PMC5409641; doi:10.3390/ijerph14040441)
Supplement: Supplementary file 1 [file ijerph-14-00441-s001.pdf]

International Journal of
Environmental Research and Public Health

MDPI

Water Access, Sanitation, and Hygiene Conditions and Health Outcomes among Two Settlement Types in Rural Far North Cameroon

Tyler J. Gorham, Joshua Yoo, Rebecca Garabed, Arabi Mouhaman and Jiyoung Lee

Supplementary materials

Table S1. Open Defecation rate and Hygiene Facility Availability.

	Village	Camp
**Open Defecation**	4 (2.6%)	15 (19.0%)
**Outdoor Latrine**	122 (79.2%)	64 (81.0%)
**Indoor Facility**	28 (18.2%)	0 (0%)

Outdoor latrine access was similar between the two study populations. No indoor facilities were reported among camp households, reflecting their nomadic herding lifestyle.

Table S2. Household-level Handwashing Rates.

How often do all family members wash their hands		Village	Camp	χ^2 *p*-value
before a meal?	Always	100.0%	83.5%	0.08
	Sometimes	0.0%	16.5%	
	Never	0.0%	0.0%	
with soap before a meal?	Always	20.8%	7.0%	0.07
	Sometimes	45.8%	21.4%	
	Never	33.3%	71.4%	
with soap after a meal?	Always	30.4%	7.1%	0.12
	Sometimes	30.4%	21.4%	
	Never	39.1%	71.4%	

"Always" responses were consistently higher among villages and "never" responses were consistently higher among camps, but the proportions of responses were not significantly different between villages and camps for any question.

Table S3. Incidence of Reported Gastrointestinal Health Conditions in Past 28 Days.

Gastrointestinal Health	Village	Camp	Fisher's Exact *p*-value
Diarrhea	3.2%	8.4%	0.12
Bloody diarrhea	1.3%	3.6%	0.34
Cramps	15.4%	8.2%	0.16

No significant difference was found between villages and camps across the three gastrointestinal health indicators.
